# Proteolysis targeting chimeras (PROTACs) are emerging therapeutics for hematologic malignancies

**DOI:** 10.1186/s13045-020-00924-z

**Published:** 2020-07-27

**Authors:** Yonghan He, Sajid Khan, Zhiguang Huo, Dongwen Lv, Xuan Zhang, Xingui Liu, Yaxia Yuan, Robert Hromas, Mingjiang Xu, Guangrong Zheng, Daohong Zhou

**Affiliations:** 1 Department of Pharmacodynamics, College of Pharmacy, University of Florida, Gainesville, FL USA; 2 Department of Biostatistics, College of Public Health & Health Professions and College of Medicine, University of Florida, Gainesville, FL USA; 3 Department of Medicinal Chemistry, College of Pharmacy, University of Florida, Gainesville, FL USA; 4 Department of Medicine, The University of Texas Health Science Center at San Antonio, San Antonio, TX USA; 5 Department of Molecular Medicine, College of Medicine, The University of Texas Health Science Center at San Antonio, San Antonio, TX USA

**Keywords:** PROTAC, Cell-specific E3 ligases, Small molecule inhibitor, Hematologic malignancy

## Abstract

Proteolysis targeting chimeras (PROTACs) are heterobifunctional small molecules that utilize the ubiquitin proteasome system (UPS) to degrade proteins of interest (POI). PROTACs are potentially superior to conventional small molecule inhibitors (SMIs) because of their unique mechanism of action (MOA, i.e., degrading POI in a sub-stoichiometric manner), ability to target “undruggable” and mutant proteins, and improved target selectivity. Therefore, PROTACs have become an emerging technology for the development of novel targeted anticancer therapeutics. In fact, some of these reported PROTACs exhibit unprecedented efficacy and specificity in degrading various oncogenic proteins and have advanced to various stages of preclinical and clinical development for the treatment of cancer and hematologic malignancy. In this review, we systematically summarize the known PROTACs that have the potential to be used to treat various hematologic malignancies and discuss strategies to improve the safety of PROTACs for clinical application. Particularly, we propose to use the latest human pan-tissue single-cell RNA sequencing data to identify hematopoietic cell type-specific/selective E3 ligases to generate tumor-specific/selective PROTACs. These PROTACs have the potential to become safer therapeutics for hematologic malignancies because they can overcome some of the on-target toxicities of SMIs and PROTACs.

## Introduction

Proteolysis targeting chimeras (PROTACs) are bivalent small molecules consisting of a ligand that binds to a protein of interest (POI) connected via a linker to a recruitment moiety for an E3 ubiquitin ligase [1]. Such conjugates can recruit the POI to the E3 ligase, promote proximity-induced ubiquitination of the POI, and lead to its degradation through the ubiquitin proteasome system (UPS). Significant progress has been made in the development of antitumor PROTACs over the last 20 years, which demonstrates that PROTACs can degrade numerous oncogenic protein targets with unprecedented efficacy [1, 2].

PROTACs are potentially more advantageous to treat tumors compared to traditional small molecule inhibitors (SMIs) in several aspects (Table 1). First, PROTACs act catalytically to induce protein degradation in a sub-stoichiometric manner [1]. Because of this unique mechanism of action (MOA), PROTACs produce longer and stronger biological effects than SMIs on a target, which allow PROTACs to be used at a much less intensive dosing regimen to be therapeutically effective than SMIs and thus reduce the risk of undesirable side effects that usually result from off-target binding of SMIs when used at higher concentrations. Second, PROTACs can be developed to target “undruggable” or difficult-to-target proteins such as transcription factors (TFs) and scaffold proteins [3]. Third, PROTACs can be used to overcome drug resistance resulting from mutations of a POI or compensatory increase in POI induced by SMIs [3]. Fourth, it is possible to achieve tumor-specific/selective degradation of a POI with PROTACs by using ligands for cell type- and/or tumor-specific/selective E3 ligases [2, 4].

**Table 1:** Comparison among PROTACs, small molecule inhibitors (SMIs), monoclonal antibodies, and therapeutic nucleic acids (TNAs)

PROTACs	SMIs	Monoclonal antibodies	TNAs
Highly selective	Poor selectivity	Selective	Selective
Oral bioavailability can be achieved	Oral bioavailability is easy to achieve	Oral bioavailability is not achievable	Oral bioavailability is not achievable
Can target proteins on cell surface and inside a cell	Can target proteins on cell surface and inside a cell	Can only target proteins on cell surface, not inside a cell	Target DNA or RNA
Tissue penetration is good	Tissue penetration is good	Tissue penetration is poor	Tissue penetration is poor
Metabolic stability is good	Metabolic stability is good	Metabolic stability is poor	Metabolic stability is poor
Sub-stoichiometric concentrations are required	Stoichiometric concentrations are required	N/A	N/A
Can target proteins without an active binding site i.e. undruggable proteins	Difficult to target proteins without an active binding site	N/A	N/A
Can target mutated proteins	Cannot target mutated proteins	N/A	N/A
Degradation blocks both enzymatic and scaffolding functions	Inhibition blocks only enzymatic functions	N/A	N/A

A number of PROTACs have been developed to target various POIs that are involved in the tumorigenesis and progression of hematologic malignancies, such as anaplastic lymphoma kinase (ALK) [5], Bcl-xL [4], BCR-ABL [6], Bruton’s tyrosine kinase (BTK) [7], BRD4 [8], CDK-6 [9], FMS-like tyrosine kinase-3 (FLT-3) [10], HDAC6 [11], and signal transducer and activator of transcription 3 (STAT3) [3]. Some of them have been shown to be extremely potent to kill leukemia and cancer cells in vitro, and can achieve complete tumor regression in vivo in animal models [3].

This review will discuss the advantages of PROTACs over other approaches for protein suppression, systematically summarize the POIs that have been targeted by PROTACs against various hematologic malignancies, and safety concerns of PROTACs and their potential of being translated into clinical applications. We further contemplate the concept of developing cell type- and tumor-specific/selective PROTACs to overcome on-target toxicities of PROTACs using the latest pan-tissue single-cell RNA sequencing (scRNA-seq) data of human adults to identify cell type-specific/selective E3 ligases [12].

## A brief history of PROTACs

In 2001, Crews’ and Deshaies’ groups developed the first PROTAC [13], which recruits the SCF^β−TrCP^ E3 ligase to degrade methionine aminopeptidase-2 (MetAp-2) [14]. In 2003, Sakamoto et al. developed PROTACs that can degrade estrogen receptor-alpha (ERα) or androgen receptor (AR) in breast cancer and prostate cancer, respectively [15]. However, these PROTACs were peptide-based and had a very high molecular weight and poor cellular permeability. To overcome these shortcomings, they developed a cell-permeable PROTAC that was able to degrade selected proteins in cells via the Von Hippel-Lindau (VHL) E3 ligase [16]. In 2008, the first small molecule-based PROTAC was developed, which utilized nutlin-3a as the mouse double minute 2 (MDM2) E3 ligase ligand to recruit MDM2 to degrade AR [17], representing a significant advancement of the PROTAC technology. From then onwards, a series of new ligands of E3 ligases such as inhibitors of apoptosis proteins (IAPs), cereblon (CRBN), and VHL were discovered and used for PROTAC development [18–20]. This was followed by several innovative strategies in the PROTAC field, including the most recent development of homo-PROTACs [21, 22] and photo-PROTACs [23, 24]. In 2013, a PROTAC based on a peptide ligand for VHL was first demonstrated to inhibit tumor growth in murine models [25]. In 2015, small molecule-based PROTACs were first reported to have in vivo activity [26, 27]. In 2019, several PROTACs that have high in vivo antitumor potency were reported [3, 4]. Recently, PROTACs against AR and ER, named ARV-110 and ARV-471, respectively, were the first to enter phase-I clinical trials (Identifier: NCT03888612; NCT04072952).

## Advantages of using PROTACs over other approaches to inhibit a POI

**Fig. 1:**
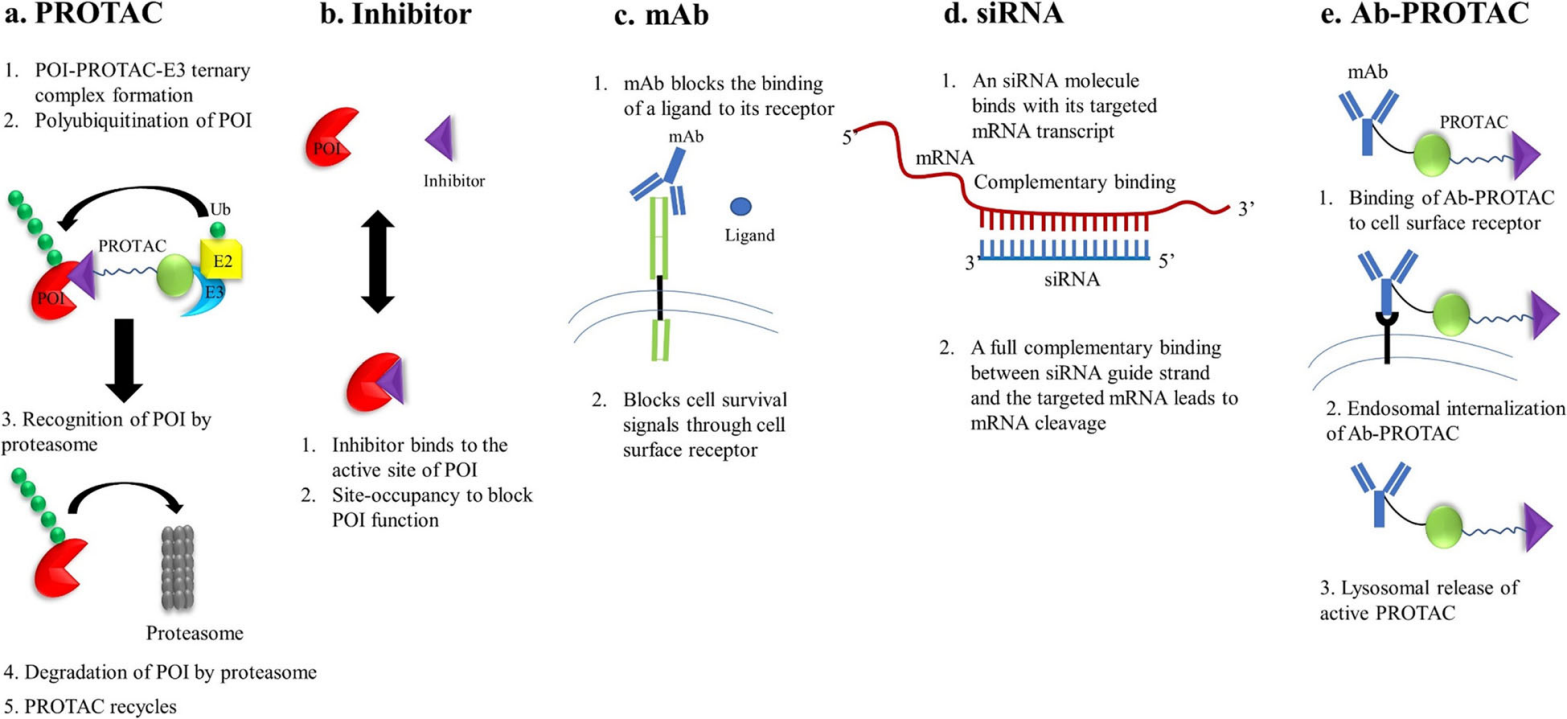
Schematic representation depicting different strategies of protein suppression. **a** PROTAC recruits an E3 ligase to a POI followed by polyubiquitination of the POI by an E2 conjugating enzyme. The polyubiquitinated POI is recognized and degraded by the proteasome. Once the POI is degraded, the PROTAC molecule can be recycled to induce the next round of POI degradation, thus working in a sub-stoichiometric manner. **b** A small molecule inhibitor (SMI) typically binds at the active site of a POI to inhibit the enzymatic functions of the POI. **c** A monoclonal antibody (mAb) binds to a cell surface receptor to block the signal transduction stimulated by a ligand, a growth factor or a cytokine. **d** siRNA binds to its targeted mRNA transcript in a complementary manner to induce mRNA cleavage and consequently translational suppression. **e** An antibody-PROTAC conjugate (Ab-PROTAC) is designed by linking a PROTAC to a mAb. Once an Ab-PROTAC binds to its cell surface receptor, it is internalized into the cytosol through endosomes. In the cytosol, an active PROTAC releases from Ab-PROTAC through lysosomal pathway which induces the degradation of POI

**Fig. 2:**
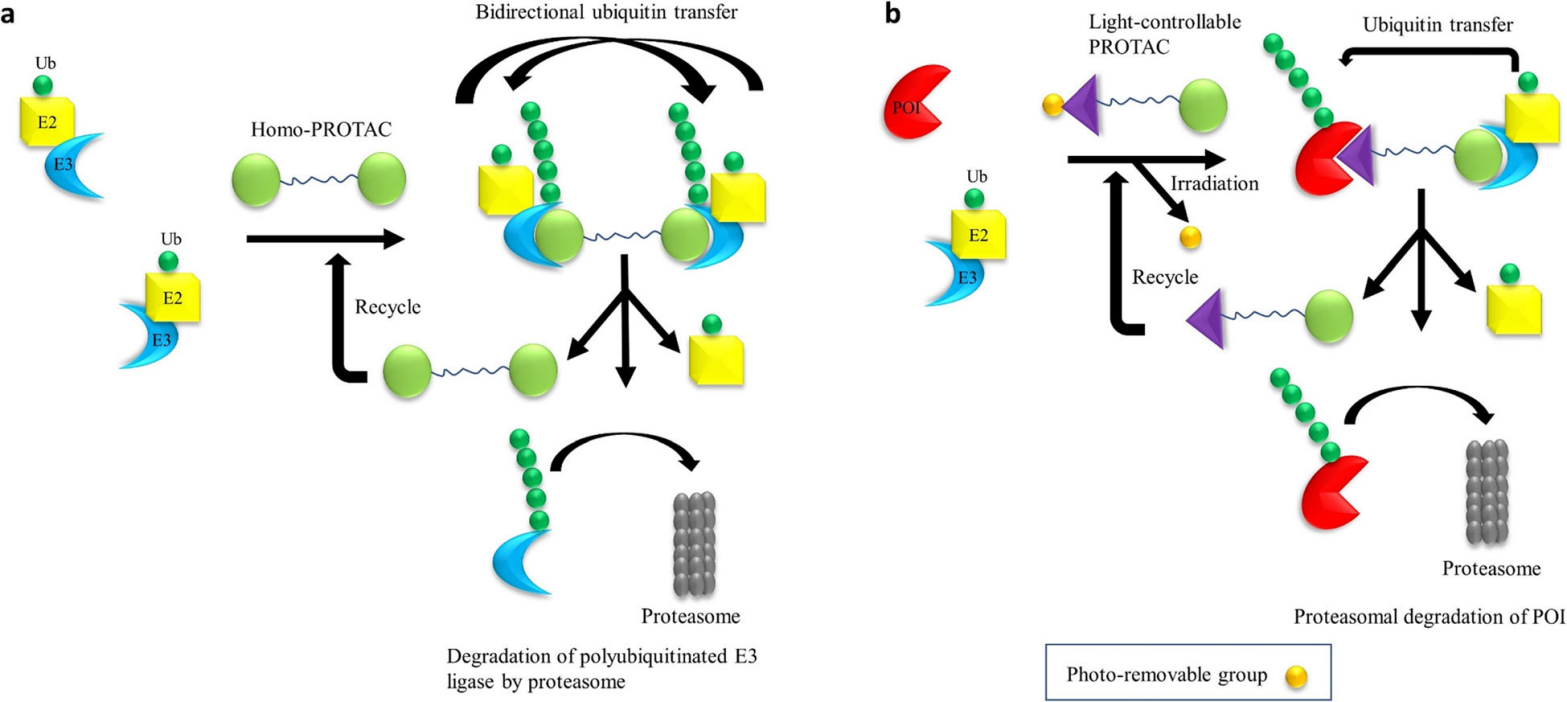
Design and mechanism of homo-PROTACs and light-controllable PROTACs. **a** Design and mechanism of homo-PROTACs. A homo-PROTAC is consisted of two E3 ligase ligand molecules connected via a linker. Homo-PROTAC recruits an E3 ligase molecule to another E3 ligase molecule followed by bidirectional polyubiquitination of E3 ligase molecules and subsequent degradation of the E3 ligase by the proteasome. **b** Design and mechanism of light-controllable PROTACs. In a light-controllable PROTAC, a photo-removable group is attached to the POI ligand or E3 ligand or linker. Upon light irradiation, the photo-removable group is detached from the light-controllable PROTAC converting it to an active PROTAC for the proteasomal degradation of POI

There are four main approaches to suppress a POI as shown in Fig. 1: (1) protein inhibition by conventional SMIs; (2) genetic manipulation to suppress a target protein, e.g., by RNAi and CRISPR-Cas9; (3) target neutralization using monoclonal antibodies; and (4) targeted protein degradation by PROTACs and other strategies. Each of these strategies has their own advantages and disadvantages. PROTACs have several potential advantages compared to the other three strategies (Table 1). The target inhibition by SMIs is the most commonly used therapeutic approach so far. The main advantage of PROTACs over SMIs is their catalytic mode of action, due to which PROTACs are required at significantly lower concentrations to exert a biological effect than SMIs. In addition, PROTACs have several other advantages including high tissue and target selectivity, and capacity to target undruggable and mutated proteins [28, 29]. Recently, homo-PROTACs were developed by tethering two E3 ligase ligands with a linker to induce self-degradation of E3 ligases [21, 22]. This approach can particularly be useful to target oncogenic E3 ligases such as MDM2. In addition, tissue/tumor-specific degradation of POIs can be achieved by using light-controllable PROTACs when combined with photodynamic therapy. In light-controllable PROTACs, a photo-removable group is attached to either a POI ligand or E3 ligand or linker. This photo-removable group is detached from the PROTAC upon light irradiation converting it to an active PROTAC [23, 30, 31]. The design and mechanisms of action of homo-PROTACs and light-controllable PROTACs are illustrated in Fig. 2. Recently, we and others have achieved tumor-selective degradation of a target protein by exploiting E3 ligases that are selectively expressed in tumors compared to normal tissues and cells such as platelets, an innovative strategy which is otherwise not achievable with conventional SMIs [2, 4, 32].

The other two strategies of target suppression, i.e., monoclonal antibodies and genetic manipulation, are limited in their scope. Thus far, monoclonal antibodies can only be used to effectively target cell surface proteins but not intracellular targets [14, 33]. On the other hand, genetic manipulation by therapeutic nucleic acids (TNAs) including antisense oligonucleotides (ASO), siRNA, miRNA, CRISPR-Cas9, etc. are still in their early developmental stage. TNAs also have several limitations including inefficient delivery to target cells, metabolic instability, and off-target effects that hinder their effective and safe use in vivo [1, 34, 35]. Some TNAs have been approved by the FDA for their use in different disease conditions, but none has been approved to treat cancers [36, 37]. Considering these limitations of using monoclonal antibodies and TNAs, PROTACs have a better chance for faster clinical development against a broader range of targets in multiple tumor types. Moreover, antibody-PROTAC conjugates (Ab-PROTACs) have been reported recently, in which a PROTAC is conjugated to a monoclonal antibody similar to antibody-drug conjugates. These Ab-PROTACs can selectively degrade POIs in targeted cells/tissues [38–40].

## PROTACs against hematologic malignancies

In this section, we systematically summarize the reported oncogenic proteins that have been targeted by PROTACs for hematologic malignancies. The relevant information of representative PROTACs against these targets including the concentration required to achieve 50% degradation (DC_50_), and in vitro and in vivo activities is shown in Table 2.

**Table 2:** Targets and efficacy of PROTACs in hematologic malignancies

				In vitro efficacy		In vivo efficacy		
Target	E3 ligase	Compound	Disease	Efficacy (EC_**50**_ or other )	Degradation (DC_**50**_/Dmax or other)	Model and efficacy	Degradation	Ref.
ALK	CRBN	TL13-112	ALCL	Karpas299 (<50 nM); SU-DHL-1 (<50 nM)	Karpas299 (DC_50_: 40 nM)	NA	NA	[41]
Bcl-6	CRBN	PROTAC 15	DLBCL	PROTAC **15** showed weak antiproliferative activity in DLBCL cell lines (>5μM)	1 μM of **15** caused 82% degradation of Bcl-6 in OCI-Ly1	NA	NA	[42]
Bcl-xL	VHL	DT2216	T-ALL	MOLT-4 (52 nM)	MOLT-4 (DC_50_: 63 nM; D_max_: 90.8%)	**MOLT-4 xenograft**: Once weekly dosing of DT2216 completely inhibited MOLT-4 xenograft **CUL76 T-ALL PDX**: Combination of DT2216 with chemotherapy drug substantially increased survival	**MOLT-4 xenograft:** Single dose of DT2216 at 15 mg/kg caused sustained reduction in Bcl-xL in MOLT-4 xenograft	[4]
BCR-ABL	VHL	SIAIS178	CML	K562 (24 nM)	K562 (DC_50_: 8.5 nM)	**K562-Luc xenograft:** SIAIS178 at 5 and 15 mg/kg/day, i.p. for 12 days dose-dependently inhibited tumor growth	**K562 xenograft:** SIAIS178 at 5 and 15 mg/kg/day, i.p. for 4 days dose-dependently depleted BCR-ABL levels in tumors 6 h after last dose	[6]
BRD4	CRBN	dBET6	T-ALL	In a set of 20 T-ALL lines, such as SUPT11(<10 nM)	MOLT4 (DC_50_< 10 nM)	**SUPT11 xenograft**: dBET6 (7.5 mg/kg, twice daily) significantly reduced leukemic burden **MOLT-4 xenograft**: dBET6 (7.5 mg/kg BID) exhibited a significant survival benefit in mice	**SUPT11 xenograft**: dBET6 led to degradation of BRD4 in leukemic bone marrow 3 h after treatment	[43]
	VHL	ARV-771	MCL	Mino (<20 nM), Z238 (~200 nM) Primary MCL cells (NA)	Mino (NA); primary MCL cells (NA)	**Z138 xenograft**: ARV-771 (30 mg/kg) was significantly more effective in improving the median and overall survival	**Z138 xenograft**: ARV-771 (30 mg/kg, daily for 5 days) resulted in the depletion of BRD4 from the spleen and bone marrow	[8]
BTK	CRBN	DD-03-171	BCL	TMD8-BTK WT (29.2 nM); TMD8-BTK C481S (128 nM); Mino (12 nM)	Effectively degraded BTK at concentrations as low as 100 nM within 4 hours of treatment in Ramos B cells	**DLBCL and MCL PDX models:** DD-03-171 at 50 mg/kg/day, i.p. for 14 days significantly inhibited the tumor burden and enhanced the survival of DFBL-96069-engrafted mice	**DLBCL and MCL PDX models:** DD-03-171 at 50 mg/kg/day, i.p. for 3 days depleted BTK levels in the spleens of DFBL-96069-engrafted mice	[44]
CDK6	CRBN	YX-2-017	Ph+ ALL	BV173 (NA); SUP-B15 (NA)	BV173 (4 nM); MUTZ-5 (NA); MHH-CALL-4 (NA); SEM (NA); Jurkat (NA)	**Ph**^**+**^**ALL xenografts**: YX-2-107 suppressed S-phase cell percentage and decreased phosphor-RB and FOXM1 expression in bone marrow cells **Ph**^**+**^**ALL PDX**: YX-2-107 suppressed peripheral blood leukemia burden	**Ph**^**+**^**ALL xenografts**: NSG mice were given YX-2-107 at 150 mg/kg per day for 3 consecutive days. YX-2-107 reduced CDK6 level in bone marrow cells	[9]
FLT-3	VHL	FLT-3 PROTAC	AML	MV4-11 (0.6 nM); MOLM-14 (NA); OCIAML-3 (>2.8 μM)	MV4-11 (NA)	NA	**MV4-11 xenograft** : FLT-3 PROTAC at 30 mg/kg daily for 3 d decreased FLT-3 by 60% in tumor	[10]
HDAC6	CRBN	12d	MM	MM.1S (EC_50_=74.9 nM, Emax=63.1%)	MM.1S (DC_50_: 1.6 nM)	NA	NA	[11]
Mcl-1	CRBN	dMCL1-2	MM	NA	OPM2 (NA) MM.1S (NA)	NA	NA	[45]
MDM2	CRBN	MD-224	ALL; AML	RS4-11 (1.5 nM); MOLM-13 (7.3 nM); MOLM-14 (10.5 nM); SIG-M5 (19.8 nM); ML-2 (4.4 nM) OCL-AML-5 (33.1 nM)	Effectively induced marked depletion of MDM2 at 1 nM in RS4-11 cells	**RS4-11 xenograft:** MD-224 at 25 mg/kg, daily, 5 days a week for 2 weeks, or at 50 mg/kg, every other day for 3 weeks achieved complete tumor regression	**RS4-11 xenograft**: Single intravenous dose of MD-224 effectively depleted MDM2 protein at 3 h, with the effect persisting for >24 h	[46]
PRC2	VHL	UNC6852	DLBCL	DB (3.4 μM) Pfeiffer (0.41 μM)	DB (DC_50_/D_max_): EED 0.61 μM/96%; EZH2 0.67 μM/94%; SUZ12 0.59 μM/82%)	NA	NA	[47]
SMARCA2/4, PBRM1PBRM1	VHL	ACBI1	AML	MV-4-11 (29 nM)	MV-4-11 cell (6 nM (SMARCA2), 11 nM (SMARCA4), 32 nM (PBRM1)), Dmax = 100% for SMARCA2/4 and PBRM1	NA	NA	[48]
STAT3	CRBN	SD-36	AML; ALCL	**AML**: MOLM-16 (0.035 μM) **ALCL**: SU-DHL-1 (0.25 μM); SUP-M2 (0.13 μM); KI-JK (0.18 μM); Karpas-299 (0.98 μM); DEL (1.48 μM);	MOLM-16 (D_max_: 90%)	**MOLM-16 xenograft**: SD-36 at 100 mg/kg weekly or 50 mg/kg twice weekly for 4 weeks induced complete tumor regression; **SUP-M2 xenograft:** SD-36 at 50 mg/kg 3 times per week completely inhibited tumor growth. SD-36 at 100 mg/kg 3 times per week achieved complete tumor regression	**MOLM-16 xenograft**: Single dose of 25 mg/kg SD-36 depleted STAT3 protein by >80% in MOLM-16 engrafted tumors; **SU-DHL-1 xenograft**: Single dose of SD-36 near-completely depleted STAT3 protein in SU-DHL-1 engrafted tumor	[3]

*ALCL* anaplastic large-cell lymphoma, *ALL* acute lymphoblastic leukemia, *AML* acute myeloid leukemia, *BCL* B cell lymphoma, *CML* chronic myelogenous leukemia, *DLBCL* diffused large B cell lymphoma, *MCL* mantle cell lymphoma, *MM* multiple myeloma, *Ph*+ *ALL* Philadelphia chromosome-positive acute lymphoblastic leukemia, *T*-*ALL* T cell acute lymphoblastic leukemia

### ALK

Anaplastic lymphoma kinase (ALK) is a receptor tyrosine kinase which is activated in many cancers including several hematologic malignancies (e.g., anaplastic large-cell lymphoma (ALCL) and diffused large B cell lymphoma (DLBCL)) and solid tumors (e.g., non-small cell lung cancer (NSCLC)) due to chromosomal translocations, substitution mutations, and gene amplification [49]. Several ALK inhibitors (crizotinib, ceritinib, alectinib, and brigatinib) have been approved for the treatment of ALK-positive NSCLC [50], and some of them are undergoing clinical trials against ALCL and other lymphomas [Identifier: NCT02465060; NCT00939770; NCT03719898]. The efficacy of ALK inhibitors is hindered by the emergence of different resistance mechanisms [50]. Researchers have adopted PROTAC technology to overcome the resistance to ALK inhibitors. The first series of ALK PROTACs were reported by Gray’s group. These PROTACs were very efficient in degrading ALK (DC_50_ ~ 10 nM in H3122 NSCLC cells) and inhibiting the proliferation of ALK-dependent ALCL and NSCLC cells. However, these PROTACs were not specific to ALK and could not degrade a mutated ALK fusion protein EML4-ALK [51]. At the same time, another group reported two ALK PROTACs (MS4077 and MS4078) that efficiently degraded ALK fusion proteins NPM-ALK and EML4-ALK in SU-DHL-1 ALCL and NCI-H2228 NSCLC cells, respectively, and potently inhibited the proliferation of SU-DHL-1 cells [5]. Another VHL-based ALK PROTAC TD-004 efficiently induced ALK degradation and inhibited the proliferation of SU-DHL-1 and H3122 cells in vitro, and reduced H3122 xenografted tumor growth in vivo [41]. Recently, a VHL-recruiting ALK PROTAC based on brigatinib, named SIAIS117, was found to be more potent than brigatinib in inhibiting the growth of G1202R mutant ALK-expressing 293T cells by inducing G1202R mutant ALK degradation [52]. The PROTACs against ALK have also been briefly discussed in a review by Kong et al. [53].

### Bcl-2 family proteins

Resistance to apoptosis plays a crucial role in tumorigenesis and is responsible for resistance to cancer therapies [54]. Therefore, targeting the apoptotic pathway becomes an attractive therapeutic strategy for cancer treatment. B cell lymphoma 2 (Bcl-2) proteins control the intrinsic mitochondria-mediated apoptotic pathway [55, 56]. SMIs targeting the anti-apoptotic Bcl-2 family proteins, including Bcl-2, Bcl-xL, and Mcl-1, have been developed for cancer treatment. Venetoclax (ABT-199), a highly selective inhibitor of Bcl-2, is the first FDA-approved Bcl-2 antagonist for the treatment of various hematologic malignancies including chronic lymphocytic leukemia (CLL) and small lymphocytic lymphoma (SLL) as a single agent, and for acute myeloid leukemia (AML) in combination with chemotherapy [57]. Wang et al. reported a Bcl-2 PROTAC with a DC_50_ of 3.0 μM in NCI-H23 lung adenocarcinoma cell line [58]; however, neither degradation nor cellular cytotoxicity data was available in hematologic tumor cell lines. Navitoclax (ABT-263), a dual inhibitor of Bcl-2 and Bcl-xL, entered clinical trials in 2006. Unfortunately, the clinically effective dosage of ABT-263 is significantly limited by thrombocytopenia because platelets solely depend on Bcl-xL for survival [59]. Recently, we reported the first example of utilizing PROTAC technology to reduce on-target toxicity of SMIs [4]. DT2216, a representative PROTAC that hijacks the VHL E3 ligase to achieve tissue/cell-selective degradation of Bcl-xL, was found to be more potent against Bcl-xL-dependent tumor cells and less toxic against platelets in comparison to conventional Bcl-xL inhibitors. Moreover, DT2216 potently degraded Bcl-xL protein and inhibited MOLT-4 T-ALL in vitro and in vivo. The discovery of DT2216 provides a proof of principle to utilize PROTAC technology to rescue undruggable targets due to previously unmanageable on-target toxicities. Using a similar strategy, we reported several PROTACs that recruited CRBN or IAPs for Bcl-xL degradation [60–63]. These Bcl-xL degraders showed significantly reduced cytotoxicity against platelets but improved killing of various tumor cells and senescent cells due to differential expression of these E3 ligases in these cells, indicating that an improved therapeutic window can be achieved by converting a non-selective SMI to a tissue/cell-specific degrader.

Mcl-1 is upregulated in various hematologic malignancies (e.g., multiple myeloma (MM) and AML) and some solid tumors (e.g., hepatocellular carcinoma and NSCLC) [64]. Tremendous efforts in drug discovery programs have generated highly selective and potent Mcl-1 inhibitors with a binding affinity at picomolar levels. Some of them (AMG-176, AMG-397, AZD5991, S64315/MIK665, and ABBV-467) have entered phase I clinical trials [65]. Notably, in late 2019, the FDA and Amgen halted the clinical trials of AMG-176 and AMG-397 due to a “safety signal for cardiac toxicity,” which is likely to be an on-target toxicity of Mcl-1 inhibition. There are reported Mcl-1 PROTACs [45, 58], dMCL1-2 as a representative, which degrades Mcl-1 in OPM2 and MM.1S MM cell lines. It is worth looking into the possibility to design tissue-specific PROTACs to reduce the on-target toxicity of Mcl-1 inhibition in the heart.

### Bcl-6

B cell lymphoma 6 (Bcl-6) is an emerging oncoprotein and therapeutic target for lymphoma. It is broadly expressed in many lymphomas and plays a significant role in lymphomagenesis by being involved in the formation of germinal centers (GCs) during the humoral immune response [66–69]. As a transcriptional repressor, Bcl-6 exerts its function by recruiting its corepressors to its *N*-terminal BTB domain to repress the expression of a broad set of genes involved in the proliferation and survival of GC B cells [70]. By targeting the interactions between the BTB domain of Bcl-6 and its corepressors, a variety of Bcl-6 inhibitors have been developed over the past decade, including peptide mimetics, small molecules, and natural compounds [71]. However, due to the challenge of disrupting PPI, these Bcl-6 inhibitors have suffered from low potency [72] and thus have limited clinical application. Chemically induced degradation of Bcl-6 by small molecules, such as BI-3802, have been found to have stronger effects than Bcl-6 inhibition and thus offer new routes to the development of lymphoma treatments [73]. By degrading Bcl-6 via the UPS, BI-3802 displayed stronger de-repression of target genes than Bcl-6 inhibitors and demonstrated antiproliferative effects in several DLBCL cell lines [73]. However, inducing Bcl-6 degradation through PROTAC strategy has met with limited success. McCoull et al. developed highly potent Bcl-6 inhibitors and converted them into PROTAC molecules by conjugating them with thalidomide [42]. Although the PROTAC molecules could degrade Bcl-6 in a number of cells, they did not improve antiproliferative activity over Bcl-6 inhibitors in DLBCL cells. Further investigation revealed that the weak antiproliferative response is due to the incomplete Bcl-6 degradation [42]. It remains unknown if complete degradation of Bcl-6 can be achieved through the exploration of the chemical space of their PROTAC molecules or through the recruitment of different E3 ligases.

### BCR-ABL

BCR-ABL is an oncogenic fusion protein implicated in the development of chronic myelogenous leukemia (CML) [74]. Tyrosine kinase inhibitors (TKIs, e.g., imatinib, dasatinib, and bosutinib) that inhibit the kinase function of BCR-ABL by competitively binding at the ATP-binding site of ABL are the standard-of-care against BCR-ABL-driven CML [75]. However, TKIs need to be administered continuously for life to treat CML patients likely due to the scaffolding functions of BCR-ABL that activate compensatory signaling pathways to promote the survival of leukemic stem cells (LSCs) [76, 77]. Since PROTACs eliminate both enzymatic and scaffolding functions of targeted proteins by inducing its degradation, they can potentially overcome the abovementioned limitations of BCR-ABL inhibitors. This led to the development of the first BCR-ABL PROTAC by Crews’ group. In this study, a lead PROTAC based on dasatinib recruiting CRBN (DAS-6-2-2-6-CRBN) degraded both ABL and BCR-ABL, whereas one recruiting VHL (DAS-6-2-2-6-VHL) could only degrade ABL. DAS-6-2-2-6-CRBN effectively reduced the viability of BCR-ABL-dependent K562 CML cells with an EC_50_ of 4.4 nM [78]. In the same year, Naito’s and Kurihara’s groups developed the specific and non-genetic IAP-dependent protein erasers (SNIPERs) against BCR-ABL. The lead SNIPERs, SNIPER(ABL)-2 and SNIPER(ABL)-3, degraded both BCR-ABL and ABL leading to growth inhibition in K562 cells at concentrations of 30 μM or higher [79]. Subsequently, Naito’s group developed a more potent BCR-ABL SNIPER, SNIPER (ABL)-39 that degraded BCR-ABL and inhibited the proliferation of several BCR-ABL-dependent CML cells at lower nanomolar concentrations [80]. Recently, Crews’ group reported the development of BCR-ABL PROTACs based on an allosteric inhibitor (GNF-5) as the warhead linked to the VHL ligand. The lead PROTAC in this study (GMB-475) induced the degradation of both BCR-ABL1 and ABL1 in a concentration- and time-dependent manner and inhibited the proliferation of K562 cells with an EC_50_ of ~ 1 μM. Interestingly, GMB-475 inhibited the proliferation of imatinib-resistant murine Ba/F3 cells expressing mutant BCR-ABL1 (T315I) with similar potency as the cells expressing wild-type BCR-ABL1 [81]. Recently, another study reported the most potent BCR-ABL PROTAC to date, named SIAIS178. SIAIS178 potently degraded BCR-ABL protein (DC_50_ = 8.5 nM) and inhibited the proliferation of K562 cells (EC_50_ = 24 nM), and further efficiently inhibited tumor growth and induced BCR-ABL degradation in K562 xenografts. More importantly, SIAIS178 was able to degrade several mutant forms of BCR-ABL [6]. More recently, light-controllable Azo-PROTACs against BCR-ABL have been reported with high potency in K562 cells [82].

### BRD4

The bromodomain and extra terminal domain (BET) family includes four bromodomain-containing proteins (BRDs): BRD2, BRD3, BRD4, and BRDT. Each BRD contains two bromodomains that serve as “epigenetic readers” to recognize acetylated lysine residues. In vitro and in vivo studies demonstrate that BET inhibitors (BETi), such as JQ1 and I-BET151, can suppress the proliferation of multiple types of tumors [83]. However, acquired resistance limits the efficacy of BETi treatment in clinical trials [84]. In addition, long-term dosing or high concentrations associated with BETi treatment also leads to obvious BRD4 protein accumulation and inefficient c-MYC suppression [85].

Recently, numerous BET PROTACs have been generated and tested in vitro and in vivo. Different E3 ligases including VHL [86–89], CRBN [43, 85, 90–93], IAP [94], MDM2 [95], AHR [96], DCAF16 [97], RNF114 [98], and RNF4 [99], were recruited to degrade BET proteins. Most of the reported PROTACs not only degrade BRD4, but also BRD2 and BRD3. However, simultaneous degradation of the three BRD proteins may increase drug toxicities [100–102]. To improve the specificity of the PROTACs, Ciulli’s group reported the first BRD4-specific PROTAC (ZXH-3-26). However, no cellular antiproliferative efficacy against cancer cells was reported for ZXH-3-26 [103].

In 2015, Bradner’s group reported the first small molecule-based BRD4 degrader, dBET1 [91]. Later, they optimized the linker and found a more potent PROTAC (dBET6) that showed better efficacy against T-ALL compared with JQ1 [43]. In the same year, Crews’ group reported a CRBN-based BET PROTAC, ARV-825, by utilizing a more potent BETi (OTX015) as the warhead for BRD4 [85]. In 2016, a VHL-based PROTAC (ARV-771) that also used OTX015 as the BRD4 warhead was generated [86]. Later, ARV-825 and ARV-771 were tested widely and displayed potent in vitro and in vivo efficacies against AML [104, 105], post-myeloproliferative neoplasm secondary AML [106, 107], MM [108], mantle cell lymphoma (MCL) [8], and DLBCL [109].

In 2018, Wang’s group reported two more potent CRBN-based BET PROTACs, BETd-260 [92] and QCA570 [93]. BETd-260 exerted high potency to rapidly degrade BRD2/3/4 and inhibit the growth of RS4-11 B cell acute lymphoblastic leukemia (B-ALL) cells in vitro and in a mouse xenograft model [92]. QCA570 is the most potent BET PROTAC that displays picomolar potency against human acute leukemia cell lines in vitro and in vivo [93]. In 2019, Crews’ group reported an MDM2-based BRD PROTAC (A1874), which not only degrades BRD4, but also stabilizes p53, and exhibited high anti-proliferative effect in several tumor cell lines including BL and myeloid leukemia [95]. Very recently, TD-428, a new BRD4 PROTAC comprised of a novel CRBN binding moiety (TD-106) and the BET binding moiety JQ1, was reported [110]. TD-428 possesses two unique properties: high specificity and lacking the Hook effect. Compared with ARV-825, TD-428 only induces moderate IKZF1/3 degradation at higher concentrations. Jiang et al. reported a new CRBN-based PROTAC (compound 15) which selectively degrades BRD2/4 over BRD3. Compound 15 suppresses the proliferation of different AML cells (MV4-11 and MOLM-13) as well as solid tumor cell lines in the NCI-60 cell line panel [111]. Interestingly, in a series of previously reported VHL-based PROTACs derived from a more potent BETi, tetrahydroquinoline (I-BET726), only selectively induce BRD3/4 degradation without any effect on BRD2, but still possess the anti-proliferation effect against AML cells [89].

### BTK

Bruton’s tyrosine kinase (BTK) is involved in constitutively active B cell receptor (BCR) signaling in various B cell malignancies CLL, DLBCL, and MCL, and thus, is an important therapeutic target in these malignancies [112]. Ibrutinib, an irreversible covalent inhibitor of BTK, is an approved drug to treat CLL and other BTK-driven cancers. Although most of the CLL patients on ibrutinib therapy show durable responses, the resistance to ibrutinib emerges due to a cysteine to serine mutation (C418S) at the ibrutinib binding site in BTK [113, 114]. Therefore, researchers have aimed to employ PROTAC technology to overcome the resistance. Using a quantitative proteomic approach, Gray’s group reported the degradation of multiple kinases including BTK by a multi-kinase degrader, TL12-186. They also reported two selective BTK degraders, one based on a promiscuous inhibitor (bosutinib) and another based on a selective BTK inhibitor (RN486). The RN486-based degrader, named DD-04-015, efficiently and selectively degraded BTK and produced a similar antiproliferative activity as RN486 in BTK-dependent TMD8 DLBCL cells. However, the ability of these PROTACs to degrade mutant forms of BTK was not evaluated [115]. Later, MT-802, a lead BTK PROTAC developed by Crews’ group, was shown to be equally effective in degrading both wild-type and C418S mutated BTK. MT-802, but not ibrutinib, was found to reduce activated-BTK levels in patient-derived CLL cells harboring the C481S mutation [116]. Using a similar strategy, Rao’s group reported a BTK degrader, named P13I, which efficiently degraded both wild-type and C418S BTK with DC_50_ values of 10 nM and 28 nM, respectively. More importantly, P13I more effectively reduced the viability of BTK-dependent wild-type and C418S BTK-expressing HBL-1 DLBCL cells compared to ibrutinib [117]. Subsequently, several potent BTK PROTACs aimed at targeting mutant BTK in ibrutinib-resistant B cell malignancies with improved cellular activities and possessing in vivo potencies were reported by different groups [7, 44, 118–120]. Recently, a light-controllable PROTAC against BTK, named pc-PROTAC3, was reported to efficiently degrade BTK in Ramos Burkitt’s lymphoma (BL) cells upon light exposure. pc-PROTAC3 was designed by attaching a bulky photo-removable group, 4,5-dimethoxy-2-nitrobenzyl (DMNB), to the imide nitrogen of a previously described BTK PROTAC MT-802 [23].

### CDK6

CDK6 is a member of cyclin-dependent kinases (CDKs) and regulates G1-S phase transition and drives cell proliferation by forming complexes with D-type cyclins to phosphorylate the tumor suppressor retinoblastoma (Rb) [121, 122]. Multiple types of leukemia cells are dependent on CDK6 for survival, including mixed-lineage leukemia (MLL) [123] and Philadelphia chromosome-positive ALL (Ph^+^ ALL) [9]. However, selectively targeting CDK6 with ATP-competitive SMIs has been challenging due to the presence of an identical ATP-binding domain in its close-related homology CDK4 [9]. Moreover, ATP-competitive inhibitors cannot disrupt the scaffolding functions of CDK6, which is important for the transcription function of CDK6 [124, 125]. Soon after the degradability of CDK4/6 and other CDK family kinase was demonstrated by Huang et al. with a multi-kinase PROTAC degrader TL12-186 [115], several groups have developed selective CDK6 PROTAC degraders by converting CDK4/6 dual inhibitor (palbociclib) to PROTAC molecules [9, 121, 126]. For example, BSJ-03-123, a CRBN-based CDK6 degrader, was reported to selectively degrade CDK6 but not CDK4 in AML cell lines because it can form a ternary complex with CDK6 and CRBN but not with CDK4 and CRBN [121]. Dominici et al. demonstrated that CDK6 degradation by PROTACs is more effective than treatment with the dual CDK4/6 inhibitor palbociclib in suppressing Ph^+^ ALL in mice [9], because PROTACs disrupted both the kinase-dependent and independent activities of CDK6. YX-2-107, a CRBN-recruiting PROTAC, degraded CDK6 selectively in Ph-like and MLL-rearranged cell lines, and inhibited their proliferation. Moreover, YX-2-107 is effective in a Ph^+^ ALL xenograft mouse model. However, the limited pharmacokinetic (PK) properties (e.g., short half-life) of YX-2-107 warrant its further optimization.

### FLT-3

FMS-like tyrosine kinase-3 (FLT-3) belongs to the class III receptor tyrosine kinases, which is activated by a FLT-3 ligand and plays a major role in the regulation of hematopoiesis [127]. Mutations in the *FLT*-*3* gene have been found in patients with ALL (1–3%), myelodysplasia (5–10%), and AML (15–35%), making it one of the most frequently mutated genes in hematologic malignancies [128]. Mutations in the *FLT*-*3* gene, such as the most common internal tandem duplication (ITD), result in its constitutive activation in these malignancies. Thus, FLT-3 has been emerged as a therapeutic target for hematologic malignancies. Several FLT-3 inhibitors have been tested in clinical trials and two (midostaurin and gilteritinib) of them have been approved by FDA for the patients with FLT3-mutated AML [129]. However, clinical response duration to FLT3 inhibitor treatment remains short partially due to the development of secondary resistance [130]. Achieving a complete and sustained inhibition of FLT-3 ITD signaling to maintain effective clinical response requires a high dose of inhibitors, which leads to off-target toxicities [131].

In an early study, using a CRBN-recruiting PROTAC based on a promiscuous kinase inhibitor to target multiple kinases failed to significantly degrade FLT-3, likely due to its poor cell permeability [115]. In contrast, a VHL-recruiting FLT-3 PROTAC from Crews’ lab potently induced FLT-3 ITD degradation in MV4-11 and MOLM-14 AML cell lines in vitro [10]. They confirmed the ability of this FLT-3 PROTAC to degrade FLT-3 in MV4-11 mouse xenografts. This study demonstrated that conversion of an FLT-3 inhibitor into a PROTAC can result in the degradation of FLT-3 ITD. However, the in vitro anti-proliferative activity of the FLT-3 PROTAC varies among AML cells compared to the FLT-3 inhibitor that was used to develop the PROTAC. For example, the PROTAC exerted a significantly better activity against MV4-11 and wild-type MOLM-14 AML cells, but was less potent in OCI-AML3 cells, than the FLT-3 inhibitor, which is likely attributed to the extent of kinase engagement/selectivity of the PROTAC [10]. In addition, both the FLT-3 PROTAC and inhibitor were ineffective against other types of hematologic malignancies, such as K562 CML cells, suggesting that the antitumor effect of the FLT-3 PROTAC on different FLT-3 ITD mutated cells is cell-dependent.

### HDAC family proteins

Histone deacetylases (HDACs) and histone acetyltransferase (HAT) are essential regulators of lysine acetylation which determines chromatin accessibility during transcription. Dysregulation of HDACs is commonly observed in both hematologic and solid malignancies, and inhibition of HDACs leads to apoptosis, differentiation, and growth arrest in tumor cells. There are 11 isoforms of zinc-dependent HDACs in humans, which can be divided into four classes: class I (HDACs 1, 2, 3, and 8); class IIa (HDACs 4, 5, 7, and 9); class IIb (HDACs 6 and 10); and class IV (HDAC 11) [132].

To date, four HDAC inhibitors (HDACi) have been approved by the FDA for the treatment of lymphomas, leukemias, and MM. Most of the approved drugs have limited isoform selectivity and none of the drug candidates have achieved clinical success for treating solid tumors as a single agent, which is probably due to their inability to achieve high concentrations in solid tumor tissues. Importantly, dose-limiting adverse effects such as cardiac toxicity associated with hERG K^+^ channel activation are hindering their progress in the clinic [133, 134].

By converting an HDACi into an isoform-selective HDAC PROTAC, it is possible to achieve improved potency and reduced off-target toxicity as reported recently [11, 135–138]. CRBN-based PROTACs recruiting a pan-HDAC inhibitor AB3 or a HDAC6 selective binder nexturastat A resulted in specific degradation of HDAC6. Wu et al. proposed that HDAC6-IKZFs dual degraders would achieve an enhanced anti-myeloma activity based on the synergistic effect of HDAC6 inhibitors and immunomodulatory imide drugs (IMiDs) [11]. Moreover, VHL-based HDAC6 degraders were capable of degrading HDAC6 without causing IKZFs degradation. HDAC6 seems to be very sensitive to PROTAC-mediated degradation, probably because of its dominant cytoplasmic localization, whereas most other isoforms are localized in the nucleus [139]. The degradation of class I HDACs was achieved by recruiting benzamide-based HDACi CI-994 as the warhead [140]. The degradation effect of this PROTAC was more significant on HDAC1-2 in comparison to HDAC3, which can probably be attributed to the HDAC binding preference of the warhead.

### MDM2

MDM2 is a known E3 ubiquitin ligase that acts as a powerful inhibitor of the tumor suppressor P53 [141]. Overexpression of MDM2 has been often observed in various human cancers and can contribute to tumorigenesis [142, 143]. Therefore, activation of the p53 pathway by antagonizing MDM2 attracts great interest for developing targeted tumor therapeutics. Several MDM2 SMIs including nutlins (nutlin 1, nutlin 2, nutlin 3) and their derivatives have been developed and exhibit good activities against various solid and hematologic malignancies, but can also cause hematopoietic suppression and gastrointestinal toxicity [144]. In addition, MDM2 inhibitor can lead to a significant compensatory increase in MDM2 expression, which can lead rapid degradation of p53 upon clearance of the MDM2 [46]. In 2018, Li et al. employed the PROTAC strategy to design small-molecule degraders of MDM2, including MD-224 as a lead degrader that targets MDM2 to CRBN for degradation [46]. MD-224 potently induced rapid degradation of MDM2 in leukemia cells. Moreover, MD-224 is capable of achieving complete and durable tumor regression in a RS4-11 xenograft model by intravenous administration. As expected, MD-224 can only inhibit the growth of leukemia cells carrying the wild-type P53 but not those with P53 mutants [46].

### PLK1

Polo-like kinase 1 (PLK1) is a Ser/Thr protein kinase which belongs to the CDC5/Polo subfamily. It is highly expressed in many different types of tumors, including hematologic malignancies such as AML and thus is an attractive tumor therapeutic targets. Mu et al. reported a CRBN-based BRD4/PLK1 dual degrader, HBL-4, which is more potent than its parental inhibitor BI2536 [145]. Interestingly, in an earlier report by the same group, a BI2536 derivative was used to build a PROTAC (compound 22f), which also displayed potent anti-proliferative activity in RS4-11 leukemia cells [146]. In that study, compound 22f only degraded BRD4. Therefore, it would be of great interest to test if compound 22f can also induce PLK1 degradation.

### PRC2

Polycomb repressive complex 2 (PRC2) is one of the two major classes of polycomb proteins, which have been broadly linked to hematologic malignancies [147–149]. The PRC2 is composed of an enhancer of zeste homolog 2 (EZH2), EED, SUZ12, RBAP46/48, and AEBP2 [150]. EZH2 acts as the core catalytic subunit of PRC2, and EED and SUZ12 subunits are indispensable for EZH2 catalytic activity [151]. As PRC2 is highly associated with hematopoietic differentiation and proliferation [152], EZH2, EED, or SUZ12 loss-of-function mutations are related to the increased activity of hematologic stem cells (HSCs) and progenitor cells [153]. High frequency of mutations in EZH2, EED, and SUZ12 are observed in patients with early T cell precursor ALL (ETP-ALL) [154, 155]. Effective inhibition of PRC2 catalytic activity has been achieved by targeting both EED and EZH2. The discovery of deazaneplanocin A (DZNep) is the first attempt to inhibit EZH2 function, which could effectively deplete EZH2, SUZ12, and EED at cellular levels [156]. Later, a series of EZH2 inhibitors were developed for targeting wild-type and/or mutated EZH2 [157]. Due to the critical role of EED in EZH2 activity, the effect of EZH2 inhibitors could be phenocopied by SMIs targeting the EED [158, 159]. Although EZH2 inhibitors are promising antitumor agents, preclinical data suggest that drug resistance could be generated by secondary mutations in both wild-type and mutant EZH2 alleles [160].

In 2020, two separate research groups successfully converted the EED inhibitors into PROTACs [47, 161]. Interestingly, both groups used the VHL ligand to design the PROTACs, but used different EED inhibitors as the warhead, as well as different strategies for linker optimization. The most potent compound named PROTAC2 developed by Bloecher’s group selectively degraded EED, EZH2, and SUZ12, and reduced the proliferation of EZH-dependent tumor cells Karpas422 [161]. The most potent compounds named UNC6852 developed by James’ group also selectively degraded EED, EZH2, and SUZ12, and potently inhibited the proliferation of DLBCL cell lines with a wild-type and Y641N mutant EZH2 [47]. Although the EED-based PROTACs could degrade EZH2, so far no selective PROTACs based on EZH2 inhibitors were reported, until 2019 when Jian et al. developed MS1943, an EZH2 inhibitor-based degrader, via hydrophobic tagging strategy, which was designed by linking an EZH2 inhibitor to a bulky adamantyl group [162]. MS1943 could effectively degrade EZH2 and selectively kill EZH2-dependent TNBC cells, whereas EZH2 inhibitors are ineffective in blocking the proliferation of TNBC cells.

### SMARCA2, SMARCA4, and PBRM1

ATP-dependent chromatin remodeling complexes (CRCs) or remodelers use the energy of ATP hydrolysis to change the packaging state of chromatin and thus regulate cellular processes related to chromatin structure such as transcription, DNA repair, DNA replication, and decatenation of chromosomes during mitosis [48, 163]. Among the four distinct families of CRCs (SWI/SNF, ISWI, CHD, INO80), SWI/SNF complexes are the most implicated in diseases with more than 20% of human cancers bearing mutations in the genes encoding mammalian SWI/SNF (mSWI/SNF) subunits [163]. mSWI/SNF complexes are separated into two forms: BRG-/BRM-associated factor (BAF) and polybromo-associated BAF (PBAF). BAF and PBAF complexes share numerous subunits, such as BRG1 (SMARCA4) and BRM (SMARCA2) ATPases, which differ by incorporating different peripheral subunits. Mutations in BAF and PBAF subunits appear to drive different malignancies [163, 164]. SMARCA4 and SMARCA2 are ATPases that are mutually exclusively present in BAF and PBAF complexes. A study found that leukemia cells rely on SMARCA4 to support their oncogenic transcriptional program [165]. Whereas SMARCA2 is essential for the growth of tumor cells that harbor loss of function mutations in SMARCA4 [166]. These findings have inspired great interest in suppressing the bromodomain of SMARCA2 and SMARCA4 through SMIs [166–168]. However, such inhibitors have failed to inhibit tumor cell growth [48] because the ATPase domain but not the bromodomain is essential for tumor cell growth [169]. By converting a non-functional bromodomain ligand to PROTAC molecules, Farnaby et al. successfully developed SMARCA2/4 degraders, including ACBI1. ACBI1 potently induced SMARCA2/4 and PBRM1 degradation in the AML cell line, and inhibited cell growth in multiple cancer cell lines [48]. Degradation of SMARCA2/4 and PBRM1 subunits may disrupt the integrity of the BAF and PBAF complexes, resulting in the dissociation of other subunits. Meanwhile, SMIs that target the allosteric binding site of the SMARCA2/4 ATPase domain and are orally bioavailable with in vivo antitumor activity have been reported [170]. However, due to the dual inhibition of SMARCA2 and SMARCA4, these allosteric inhibitors have a dose-limiting tolerability issue that hampered their further exploration. It would be interesting to see if converting these dual SMARCA2 and SMARCA4 inhibitors to PROTACs would result in the selective degradation of SMARCA2 or SMARCA4.

### STAT3

Signal transducer and activator of transcription 3 (STAT3) belongs to the STAT family of proteins, which are both signal transducers and TFs [171]. Aberrant activation of STAT3 is associated with many human malignancies [172–174], because STAT3 regulates numerous genes involved in tumorigenesis, progression, and drug resistance, and thus it has become an attractive cancer target [172]. Several SMIs that block the dimerization of STAT3 have reached the clinical development stage, but only exhibited very limited clinical activity [173]. One major reason for their ineffectiveness is that monomeric STAT3 also has transcriptional activity [175]. To overcome the shortcoming, Wang’s group developed a series of STAT3 PROTACs, including SD-36 [3]. SD-36 efficiently degraded STAT3 protein in MOLM-16 AML cells and several anaplastic ALCL cell lines (SU-DHL-1, DEL, and KIJK). Interestingly, although SD-36 also binds to other STAT proteins, such as STAT1 and STAT4, it only degrades STAT3. In addition, SD-36 effectively degraded mutated STAT3 proteins in tumor cells. Further, SD-36 had desirable PK/pharmacodynamic (PD) properties, was well tolerated and achieved complete and long-lasting tumor regression in mice xenografted with AML and ALCL cell lines. More examples of STAT3 degraders were found in another study from Wang’s lab [176]. This study provides an excellent example that PROTAC technology can be used to target a previously undruggable target such as STAT3. However, STAT3 PROTACs may have some limitations in targeting leukemia than lymphoma, because SD-36 demonstrates potent activity in five of nine lymphoma cell lines, while showing activity in only one of nine AML cell lines [3]. Developing PROTACs that can target other STAT family proteins may add an additional layer of benefits in the treatment of hematologic malignancies.

### Other targets

In addition to the above targets, there are also some less studied PROTAC targets (SIRT2, PCAF/GCN5, BRD7, BRD9, IRAK4, TRIM24, and RIPK2) that are implicated in various malignancies, including leukemia and lymphoma. Schiedel et al. reported a PROTAC against sirtuin 2 (SIRT2). This PROTAC selectively induced SIRT2 degradation in HeLa cells and led to more pronounced acetylation of the microtubule network than the corresponding inhibitor [177]. P300/CBP-associated factor (PCAF) and general control nonderepressible 5 (GCN5) are multidomain epigenetic proteins, containing an acetyltransferase and a bromodomain, which are involved in many cellular processes including proliferation, DNA damage repair, and inflammation [178]. A PCAF/GCN5 PROTAC, named GSK983, efficiently degraded PCAF and GCN5 in THP1 acute monocytic leukemia cells [179]. BRD7 and BRD9 are bromodomain-containing subunits of the BAF and PBAF complexes, respectively [164]. Brander’s group reported a selective degrader of BRD9, named dBRD9, which efficiently degraded BRD9 and exerted a 10–100-fold increase in antiproliferative activity than the corresponding inhibitor against MOLM-13 and other AML cell lines [180]. Subsequently, Ciulli’s group reported a VHL-based dual BRD7 and BRD9 degrader, VZ185, which was found to be more effective than the corresponding inhibitor in reducing the viability of EOL-1 acute myeloid eosinophilic leukemia cells and A-204 malignant rhabdoid tumor cells [181]. The hyperactivation of interleukin-1 receptor-associated kinase 4 (IRAK4), a key mediator of innate immunity, is linked with autoimmune diseases and cancer [182]. IRAK4 has both enzymatic (kinase-dependent) and scaffolding (kinase-independent) functions [183]. Nunes et al. reported a VHL-based PROTAC that was capable of degrading IRAK4 in peripheral blood mononuclear cells and dermal fibroblasts with DC_50_ values of 151 nM and 36 nM, respectively [184]. In addition, PROTACs targeting tripartite motif-containing protein 24 (TRIM24), receptor-interacting protein kinase 2 (RIPK2), and the Janus kinase family enzymes for degradation may also have the potential to be used to treat certain types of hematologic malignancies but require further studies [185–187].

## Potential oncoproteins that can be targeted by PROTAC in hematologic malignancies

### CALR

*Calreticulin* gene (*CALR*) mutations frequently occur in Philadelphia chromosome (Ph)-negative myeloproliferative neoplasms (MPNs), including essential thrombocythemia (ET), primary myelofibrosis (PMF), and polycythemia vera (PV) [188–190]. *CALR* mutations are mutually exclusive with *JAK2* or *MPL* mutations in MPNs, suggesting common oncogenic pathways. *CALR* mutations are located in exon 9 and generate a + 1 base-pair translation frameshift. Although the extent of the C-terminal alterations vary among *CALR* mutations, all resulting variants share a loss of C-terminal 27 amino acids with a concomitant gain of a novel peptide consisting of 36 amino acids.

*CALR* mutations has been shown to be pathogenic and serve as a disease-initiating event to favor the expansion of the megakaryocytic lineage by specific activation of the thrombopoietin receptor and uncontrolled activation of the JAK2/STAT pathway [191, 192]. The oncogenic CALR mutant protein could be an excellent PROTAC target to treat MPN patients with *CALR* mutations. It would be ideal to design the PROTAC against all CALR mutant proteins using specific ligand for the unique 36 amino acids in the C-terminal of all CALR mutants and a megakaryocytic lineage specific E3 ligase.

### NPM1

*Nucleophosmin* (*NPM1*) is one of the most commonly mutated genes in AML, occurring in approximately 50% of adult and 20% of childhood AML with normal karyotypes [193, 194]. Due to its clinical significance, *NPM1*-mutated AML was categorized as a distinct leukemic entity in the WHO-2016 classification [195]. *NPM1* mutations are heterozygous and restricted to exon-12, causing a frameshift to eliminate the nucleolar localization sequence of NPM1 and to generate a new C-terminal nuclear export signal motif. Therefore, mutations in *NPM1* result in mislocalization of NPM1 mutant proteins to the cytoplasm (NPM1c^+^). NPM1c^+^ acts as a dominant negative mutant for NPM1 function and has a gain-of-function role in pathogenesis of the AML phenotype [196, 197]. Although there are many different strategies in targeting NPM1 such as inhibition of translocation of NPM1c^+^ [198] and proteasomal degradation of NPM1c^+^ [199], the unmet needs to develop effective drugs targeting NPM1 is still growing. The development of a PROTAC specifically targeting NPM1c^+^ could be a superb strategy. Giving the distinct cellular localization of WT NPM1 and NPM1c^+^, a cytosolic E3 ligase would effectively eliminate the degradation of WT NPM1, if the ligand is not specific enough to NPM1c^+^. In addition, efforts could be directed to screen specific ligands for type A *NPM1* mutation, which accounts for ~ 80% of the *NPM1* mutations and has a unique 12 amino acid peptide in the C-terminal including a lysine.

### Oncofusion proteins

Chromosomal translocations are very common in hematologic malignancies [200, 201]. It is well established that chromosomal translocations promote the development of hematologic malignancies either through the formation of oncogenic fusion protein or through oncogene activation by a stronger promoter or enhancer [200, 201]. Both the “*alien*” oncofusion proteins and the miss-activated oncoproteins are potential PROTAC targets. Indeed, several misactivated oncoproteins such as Bcl-2 and Bcl-6, and oncofusion proteins such as BCR-ABL have been targeted by PROTACs for specific subtypes of hematologic malignancies (see above sections in this review). Theoretically, these oncofusion proteins could be ideal PROTAC targets, since they are “*alien*” to human cells and the side effects would be easier to manage. The most challenging step for such PROTAC development will be the screening for relative specific ligands for such oncofusion proteins. The available crystal structures for some of these oncofusion proteins will aid this endeavor. The oncofusion proteins are often specific for subtype of hematologic malignancies, which also have potential value for PROTAC development in terms of E3 ligase selections, e.g., there are four most prevalent oncofusion proteins in AML: PML-RARα, AML1-ETO, CBFβ-MYH11, and MLL-fusions, while ETV6-RUNX1 and TCF3-PBX1 fusions are common in B-cell ALL [202].

## Safety concerns of PROTACs

As discussed above, PROTACs have several advantages over SMIs [32]. It is encouraging that the PROTACs targeting AR and ER have advanced to the clinic and the preliminary results from these clinical studies reveal that the AR PROTAC ARV-110 appears clinically effective in a patient with metastatic castration-resistant prostate cancer (https://ir.arvinas.com/node/7666/pdf). However, there are still some safety concerns about PROTACs, which should be considered before advocating for their clinical translation. Although PROTACs can be more potent than SMIs against a POI, they might produce greater on-target toxicities than SMIs for several reasons. First, SMIs rarely inhibit the functions of their targets completely, while PROTACs can almost entirely deplete their targets. The incomplete inhibition of a POI with SMIs may be tolerable [203], while their complete depletion with a PROTAC might be detrimental if the POI is essential for normal cell function. Second, some POIs have both enzymatic and scaffold functions. The latter may be important for normal cell functions. In this case, in contrast to SMIs which only inhibit enzymatic function, PROTACs can completely deplete the protein, resulting in the loss of both enzymatic activity and scaffolding function, which can potentially cause some undesirable consequences. Third, SMIs may cause a transient inhibition of a protein function while PROTACs induce prolonged depletion of the target protein due to their unique MOA. The prolonged depletion of a target by PROTACs may cause on-target toxicities if a POI is indispensable for normal functions [4].

In addition, many PROTACs are not completely selective and can degrade proteins other than their desired targets [7, 90, 204, 205]. For example, proteins that are not directly bound to a PROTAC can be degraded if they are a part of the same complex or are in close proximity with the target protein [161]. An off-target effect can also arise from the neomorphic interactions with neosubstrates of an E3 ligase if the E3 ligase ligand used to generate the PROTAC or the PROTAC itself can bind the neosubstrates [206]. A well-characterized example of off-target activities of PROTACs are CRBN-based PROTACs because CRBN ligands, including thalidomide, pomalidomide, and lenalidomide, used for the CRBN-targeted PROTACs can recruit several neo-substrates such as SALL4, IKZF1, and IKZF3 to CRBN to induce their degradation [207, 208]. This can lead to some adverse effects including teratogenic effect and cardiovascular, hepatic, and neuronal toxicities. Similarly, the VHL E3 ligase is essential for the ubiquitination and degradation of hypoxia-inducible factor-1 alpha (HIF1a) [209], a TF that regulates many genes necessary for tumor growth. If VHL is overly engaged by a PROTAC, it can potentially inhibit the degradation of HIF1a to promote tumorigenesis. Therefore, profiling the changes in global protein expression by proteomics will be helpful to appreciate the potential off-target toxicities of PROTACs [3, 4, 43]. In addition, the formation of a stable ternary complex of the POI, PROTAC, and E3 ligase is essential for PROTACs to induce specific degradation of the POI. However, high concentrations of PROTACs produce the “Hook effect,” which may lead to the formation of binary complexes of PROTACs and E3 ligases [28]. These binary complexes can potentially recruit off-targets and lead to their degradation and off-target toxicities*.* Lastly, the UPS maintains the homeostasis of intracellular proteins [210]. If PROTACs cause disruption of the UPS homeostatic function, they can cause some safety concerns for clinical application. Therefore, strategies should be developed to reduce both the on-target and off-target toxicities of PROTACs in order to more rapidly translate PROTACs into the clinic. In the following sections, we will focus our discussion on the strategies that can potentially be used to reduce the on-target toxicities of PROTACs.

## Cell type- and tumor-specific/selective PROTACs

One of the most important safety concerns about PROTACs discussed above are on-target toxicities, which may be avoided to a large extent by utilizing ligands against cell type- and tumor-specific/selective E3 ligases for designing PROTACs [2]. These PROTACs are expected to avoid the on-target degradation of POIs in cells or tissues where the specific/selective E3 ligases are not expressed. Although numerous PROTACs have been generated in the past few years, only a few of them are tumor-selective to avoid normal tissue toxicity, including platelet sparing Bcl-xL PROTACs reported by us recently by taking advantage of the E3 ligases that are barely expressed in platelets [4, 60–63]. These findings are in agreement with Crews’ recent suggestion that developing tumor-specific/selective PROTACs has the potential to reduce the on-target toxicities of PROTACs [2].

There are several strategies to achieve tumor-specific/selective degradation of tumor-associated POIs by PROTACs as discussed in a recent review [211]. First, if a POI is tumor-specific, we can generate a tumor-specific PROTAC by using any available E3 ligases in the tumor tissues to degrade this POI. One example is BCR-ABL1 PROTACs because BCR-ABL1 is specifically expressed in CML cells [81]. This strategy may be applicable to many of the oncofusion proteins specifically expressed in leukemia cells. Second, if a POI is not tumor-specific but is specific for a tumor-derived tissue or cell, we can still develop tumor-selective PROTACs by targeting the POI to any available E3 ligases in that tissue or cell, providing the POI is dispensable for the normal tissue function or the normal tissue function is nonessential. An example is BTK, that is important for B cell development and can promote the development of B cell lymphoma [118]. Third, if a POI is not tissue/tumor-specific, we can generate a tumor-selective PROTAC by targeting this POI to a tissue- and/or tumor-selective E3 ligase to limit its on-target toxicity. Bcl-xL PROTACs discussed above are an example of this approach [4, 61–63]. However, numerous POIs are ubiquitously expressed in both tumor and normal tissues and are indispensable for normal cell functions. To overcome the on-target toxicity of a PROTAC, targeting this kind of protein in tumor cells will require the identification of tumor-specific/selective E3 ligases [2, 211]. This can be accomplished by profiling E3 ligase expression at a cellular level as discussed below.

## Identification of cell type-specific/selective E3 ligases

The recent advancement in the single-cell RNA-Seq (scRNA-Seq) technology has allowed us to profile gene expression at a single-cell level and deconvolute the cell-type heterogeneity in tissues. By leveraging a recent human pan-tissue scRNA-Seq data (a.k.a. Human Cell Landscape; HCL) [12], we can identify cell-type specific/selective E3 ligases to facilitate the development of tumor-specific/selective PROTACs. A detailed description of the HCL data, including sample preparation, data sequencing procedure, and batch removal were previously described [12]. We obtained the data of the batch-removed counts from https://figshare.com/articles/HCL_DGE_Data/7235471, including 37 tissues from human adults and a total of 315 different cell types, and employed R package *Seurat* for data processing and quality control [212]. To be more specific, the cells with low quality (i.e., unique transcript counts less than 300) or exhibiting an aberrantly high gene count (i.e., unique transcript counts over 2500), or those with mitochondrial contamination (i.e., > 20% mitochondrial counts) were filtered out. The transcript expression measurements of each cell were normalized against total expression using a global-scaling normalization method, followed by log-transformation. Since multiple cell samples existed in each cell type of a tissue, we calculated the mean expression value by taking their averages. Finally, transcripts for 574 from more than 600 putative E3 ligases were successfully identified in the HCL data [213]. The resulting transcript expression levels of the E3 ligases were visualized by heatmap [214], and presented in Additional file 1. Information including tissue name, cell types, and gene expression levels corresponding to Additional file 1 are provided in Additional file 2. There is an obvious cluster of cell-type non-specific E3 ligases consisting of ZFAND5, WSB1, and TNFAIP3, because they are expressed across different cell types in many tissues. Some E3 ligases are considered cell-type specific/selective because they are highly expressed in certain cell types. For example, PEX10, KBTBD4, PATZ1, RABGEF1, AREL1, LONRF2, FBXL16, ZBTB45, KCNB1, RNF2208, KCTD16, KCTD4, DPF1, MKRN3, and RHOBTB1 are highly expressed in neurons of the temporal lobe but lowly expressed in most cell types in other tissues; KRT8 is highly expressed in cells in gastrointestinal tract, prostate, and pancreas, but lowly expressed in cells of brain and hematopoietic system (Additional file 1).

**Fig. 3:**
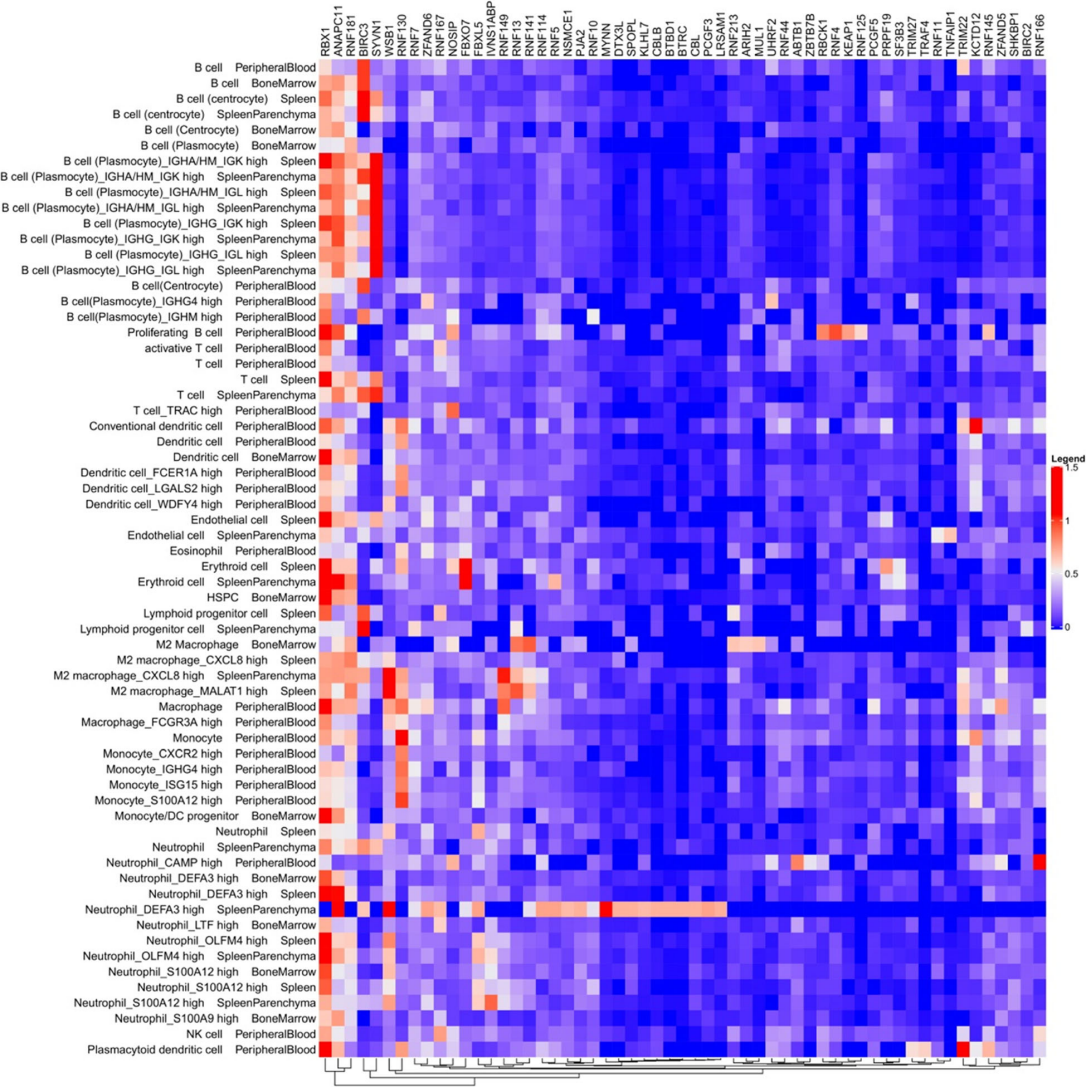
Heatmap of single-cell expression of 57 E3 ligases in hematopoietic tissues. The levels of single-cell expression of 57 E3 ligases in different types of hematopoietic cells from the peripheral blood, bone marrow, and spleen in human adults were abstracted from the Human Cell Landscape (HCL) data. In the heatmap, each row represents a cell type, and each column represents an E3 ligase. The color legend shown on the right was determined by averaging the expression values of all cells within a specific cell type. These E3 ligases were clustered by the hierarchical clustering algorithm, and the resulting dendrogram was shown at the bottom. The 57 E3 ligases presented in the figure were chosen according to the criteria: E3 ligases with an average expression value > 0.5 in at least one cell type. Single cell RNA-sequencing (scRNA-seq) data were from the website: https://figshare.com/articles/HCL_DGE_Data/7235471.

Furthermore, we analyzed the single-cell gene expression profile of E3 ligases in the hematopoietic system to identify E3 ligases differentially expressed in different types of hematopoietic cells (Additional file 3). The top 57 highly expressed E3 ligases in hematopoietic cells are presented in Fig. 3. Among them, RBX1, ZFAND5, WSB1, ANAPC11, SOCS3, KRT8, RNF181, LGALS3BP, BIRC3, and IVNS1ABP are the top 10 ubiquitously expressed E3 ligases in various hematopoietic cells, of which RBX1 [215], ZFAND5 [216], WSB1 [217], ANAPC11 [218], SOCS3 [219], RNF181 [220], and BIRC3 [221] have been reported to have E3 ligase activity.

Of these highly expressed E3 ligases in hematopoietic cells, BIRC3 (or cIAP2) is of particular interest for the design of hematopoietic cell-selective PROTACs. BIRC3 belongs to the IAP family proteins, of which cIAP1 and XIAP have been widely used to develop protein degraders named SNIPERs [18, 79, 80, 222–224]. Recently, we also developed IAP-recruiting PROTACs targeting Bcl-xL for degradation in lymphoma and a set of solid tumor cell lines [61]. BIRC3 has been reported to have E3 ligase activity [221], but has not been used to design PROTACs. This is likely attributable to the lack of specific ligands for BIRC3 [225, 226], as well as overlooked by its low expression in most tumor cells (detectable in only 8% of 60 tumor cell lines) compared to other IAP proteins, such as cIAP1 and XIAP [227]. Interestingly, although the levels of BIRC3 are low in the majority of cancers, it is highly expressed in many hematologic malignant cells, such as CLL, MCL, follicular lymphoma (FL), marginal zone lymphoma (MZL), splenic marginal zone lymphoma (SMZL), and DLBCL, compared to the other members of the IAP family proteins [228, 229]. We also found that BIRC3 is highly expressed in T cell lymphoma (TCL) cells compared to other IAP proteins [61]. Therefore, BIRC3 represents a promising E3 ligase that can be used to design cell-selective PROTACs targeting hematologic malignancies, especially TCL. Another advantage to using IAPs to design PROTACs is that their ligands are also able to kill hematologic malignant cells because some of them depend on IAPs for survival [230]. The shortcoming of IAP-recruiting PROTACAs is the induction of autoubiquitination and degradation of some IAP proteins such as cIAP1 [18]. This drawback can be avoided by modifying the linker attachment site of the E3 ligase ligand MeBS [231]. Compared to cIAP1, BIRC3 is relatively less sensitive to the ligand-induced autoubiquitination and degradation and thus may be more suitable for the design of PROTACs [225].

In addition to BIRC3, we found that SYVN1 (also known as HRD1) is a plasmocyte selective E3 ligase. SYVN1 is lowly expressed in proliferating B cells and moderately expressed in some differentiated B cells, i.e., centrocytes, but highly expressed in plasmocytes (Fig. 3), suggesting that its expression is upregulated during B cell differentiation. In addition, the expression of SYVN1 is low in other cell types in the hematopoietic system (Fig. 3). SYVN1 was originally identified as an E3 ligase within an endoplasmic reticulum (ER) membrane-anchored complex that targets luminal misfolded glycoproteins for degradation [232, 233]. It is a key regulator for B cell proliferation and development [234]. Loss of SYNV1 in B cell precursors leads to a severe developmental block at the transition from large to small pre-B cells [234]. SYVN1 can also regulate B cell immunity by protecting B cells from activation-induced cell death via degrading the death receptor Fas [235]. Moreover, SYVN1 can also mediate the degradation of P53 [236]. Therefore, SYVN1 has been implicated in the oncogenesis of activated B cell-like DLBCL (ABC-DLBCL) [237], and has been considered a potential therapeutic target for restoring the tumor suppressor activity of unstable lymphoma-associated Blimp-1 mutants [237]. In addition, MM cells express high levels of SYVN1 to cope with ER stress resulting from immunoglobulin hyperproduction [238]. Therefore, it would be of great interest to determine whether SYVN1 can be used to design cell type-specific PROTACs to target hematologic malignancies associated with B cells, particularly MM.

Besides the E3 ligases discussed above, there are many other cell type-specific E3 ligases identified at the single-cell level (Fig. 3), such as KCTD12 in blood dendritic cells, and TRIM22 in blood plasmacytoid dendritic cells. Gaining insight into the function and designing ligands for these cell type-specific E3 ligases would be greatly useful for the development of cell type- and/or tumor-specific/selective PROTACs in the future.

## Clinical translation of PROTACs

To date, two PROTACs have entered into clinical trials for the treatment of prostate and breast cancers. As discussed above, although numerous PROTACs have been developed to target oncoproteins associated with hematologic malignancies, none has entered into the clinical trials for this indication thus far. In this section, we will briefly discuss the PROTACs currently undergoing in clinical trials, and those in preclinical development for the treatment of hematologic malignancies.

### PROTACs in clinical trials

ARV-110 is an orally bioavailable AR PROTAC that entered into a clinical trial to treat patients with metastatic castrate-resistant prostate cancer (NCT03888612) in 2019. Recently, ARV-110 was reported to have an acceptable safety profile and appears clinically effective in a patient with metastatic castration-resistant prostate cancer (https://ir.arvinas.com/node/7296/pdf). ARV-471 is a PROTAC designed to specifically target ER. In preclinical studies, ARV-471 demonstrated near-complete ER degradation in tumor cells, and induced robust tumor shrinkage when dosed as a single agent in multiple ER-driven xenograft models. ARV-471 is currently in a phase I clinical trial (NCT04072952). Its safety, tolerability, pharmacokinetics, and anti-tumor activity in patients with ER^+^/HER2^−^ locally advanced or metastatic breast cancer (https://ir.arvinas.com/node/7296/pdf) will likely be reported in the coming months. A positive result from these PROTAC clinical studies will provide proof-of-principle that PROTACs can be developed as effective antitumor therapeutics.

### PROTACs in preclinical development for hematologic malignancies

Although PROTACs have been developed to successfully target a number of targets associated with hematologic malignancies, none has entered into the clinical trials. However, several PROTACs have shown promising preclinical results in the treatment of hematologic malignancies [3, 4, 44], and will likely be evaluated in the clinic in the near future. In addition, many pharmaceutical companies have invested heavily in the development of their antitumor PROTAC pipelines. For example, Nurix is going to file an IND application with the FDA at the end of this year to test its BTK PROTAC in patients with B cell malignancies (https://www.nurixtx.com/research-development/pipeline/), Kymera is advancing a STAT3 PROTAC as one of their drug candidates in oncology (https://www.kymeratx.com/pipeline/), and Dialectic Therapeutics (DT) is pursuing preclinical development of the Bcl-xL PROTAC DT2216 for the treatment of hematologic malignancies and solid cancer tumors in 2021 (https://www.dtsciences.com/#science). Therefore, we expect to witness more clinical studies on PROTACs for hematologic malignancies in the near future.

## Conclusions and perspectives of PROTACs as therapeutics for hematologic malignancies

Targeted protein degradation using PROTACs has emerged as a novel therapeutic modality in drug discovery. Since the first PROTAC was reported, researchers have made remarkable progress in the field in the last 20 years. PROTACs have been shown to have unique advantages over conventional SMIs. However, the high molecular weight and complex structure of PROTACs makes some of the PROTACs have low in vivo efficacy. Cellular permeability, compound stability, tissue distribution, and other pharmaceutical properties need to be balanced during PROTAC optimization to generate a desirable therapeutic. In addition, PROTACs based on VHL and CRBN have some drawbacks associated with tumor-suppressor functions of these proteins. For example, VHL is frequently mutated in kidney cancer [239, 240]; therefore, VHL-based PROTACs cannot be used to treat kidney cancer patients with mutation or deletion in *VHL* gene. Moreover, resistance to both VHL- and CRBN-based PROTACs was reported in cancer cells following chronic treatment, which was primarily led by genomic alterations that compromise core components of the relevant E3 ligase complexes [241]. Also, careful optimization of dosing is needed to avoid tumor suppressor functions of VHL and CRBN.

To date, two PROTACs targeting AR and ER are already in phase I clinical trials to treat patients with prostate and breast cancers, respectively. Several PROTACs have shown promising preclinical results in the treatment of hematologic malignancies [3, 4, 46], which have a great potential of being evaluated in clinical trials in the near future. In the translational applications of PROTACs, safety concerns should be taken into consideration. Identifying more strategies to develop safer PROTACs will have greater potential for PROTACs to be successful in the clinic. The development of tumor-specific/selective PROTACs based on cell-type and tumor-specific/selective E3 ligases may revolutionize the field of PROTACs as antitumor therapeutics. Breakthroughs in scRNA-seq have greatly enhanced our ability to determine cell-type specific transcriptomes [12], and enable us to understand the profile of oncogenic targets and E3 ligases at the single-cell level. Based on this, we can choose more specific E3 ligases to target POIs to improve the efficiency of PROTACs and maximally reduce the toxicities to normal cells, which may be particularly applicable for hematologic malignancies.

Identifying appropriate ligands for E3 ligases and substrates is the most critical but challenging step for the design of tumor-specific/selective PROTACs. Among the hundreds of E3 ligases in the human genome, only a few have been used in PROTACs, Therefore, more candidate E3 ligases should be further explored to expand the choices for the rational design of PROTACs. In addition, recently researchers have developed similar strategies to induce targeted degradation of RNAs (e.g., oncogenic micro-RNAs) by recruiting nucleases using small-molecules. These RNA degraders are named as ribonuclease targeting chimeras (RIBOTACs), which also have the potential to be developed as novel anticancer therapeutics [242, 243].

## Supplementary information

**Additional file 1.** Profiling E3 ligase expression at single cell level in different tissues. The heatmap shows the single-cell expression levels of 574 E3 ligases across all cell types and tissues in human adults from the Human Cell Landscape (HCL) data. In the heatmap, each row represents a cell type, and each column represents an E3 ligase. The color legend was shown on the right, which was determined by averaging the expression values of all cells within a tissue-specific cell type. These E3 ligases and the different cell types were clustered by the hierarchical clustering algorithm, and the resulting dendrograms were shown on bottom and right, respectively.

**Additional file 2.** List of 574 E3 ligases expressed in different cell types from all tissues.

**Additional file 3.** List of 574 E3 ligases expressed in different types of hematopoietic cells in the blood, spleen, and bone marrow.

## Data Availability

All data and materials supporting the conclusion of this study have been included within the article and the additional files.

## Abbreviations

ABC-DLBCL: Activated B cell-like diffused large B cell lymphoma
Ab-PROTACs: Antibody-PROTAC conjugates
ADC: Antibody-drug conjugate
ALK: Anaplastic lymphoma kinase
AML: Acute myeloid leukemia
AR: Androgen receptor
BTK: Bruton’s tyrosine kinase
BAF: BRG-/BRM-associated factor
Bcl-2: B cell lymphoma 2
Bcl-6: B cell lymphoma 6
BET: Bromodomain and extra terminal domain
BETi: Bromodomain and extra terminal domain inhibitor
BL: Burkitt’s lymphoma
BRDs: Bromodomain-containing proteins
CALR: Calreticulin
CDK: Cyclin-dependent kinase
CLL: Chronic lymphocytic leukemia
CML: Chronic myelogenous leukemia
CRBN: Cereblon
CRC: Chromatin remodeling complex
DMNB: 4,5-Dimethoxy-2-nitrobenzyl
DLBCL: Diffused large B cell lymphoma
DZNep: 3-Deazaneplanocin A
ERα: Estrogen receptor-alpha
ET: Essential thrombocythemia
ETP-ALL: Early T cell precursor ALL
EZH2: Enhancer of zeste homolog 2
FLT-3: FMS-like tyrosine kinase-3
GC: Germinal center
GCN5: General control nonderepressible 5
HAT: Histone acetyltransferase
HDACi: Histone deacetylase inhibitor
HDAC: Histone deacetylase
HIF1a: Hypoxia-inducible factor-1 alpha
HSC: Hematologic stem cell
IMiDs: Immunomodulatory imide drugs
IRAK4: Interleukin-1 receptor-associated kinase 4
LSC: Leukemic stem cell
MDM2: Mouse double minute 2 homolog
MCL: Mantle cell lymphoma
MLL: Mixed-lineage leukemia
MM: Multiple myeloma
MPNs: Myeloproliferative neoplasms
NPM1: Nucleophosmin
NSCLC: Non-small cell lung cancer
PCAF: P300/CBP-associated factor
PD: Pharmacodynamic
PDX: Patient-derived xenograft
PK: Pharmacokinetic
PLK1: Polo-like kinase 1
PROTACs: Proteolysis targeting chimeras
RIPK2: Receptor-interacting protein kinase 2
PBAF: Polybromo-associated BAF
Ph^+^ ALL: Philadelphia chromosome-positive acute lymphoblastic leukemia
PMF: Primary myelofibrosis
PV: Polycythemia vera
SCLC: Small cell lung cancer
scRNA-seq: Single-cell RNA sequencing
SIRT2: Sirtuin 2
SLL: Small lymphocytic lymphoma
SMIs: Small molecule inhibitors
SNIPER: Non-genetic IAP-dependent protein eraser
STAT3: Signal transducer and activator of transcription 3
T-ALL: T cell acute lymphoblastic leukemia
TCL: T cell lymphoma
TF: Transcription factor
TNAs: Therapeutic nucleic acids
TRIM24: Tripartite motif-containing protein 24
UPS: Ubiquitin proteasome system
VHL: Von Hippel-Lindau
