# Supplementary material for: Proteolysis targeting chimeras (PROTACs) are emerging therapeutics for hematologic malignancies
Source: J Hematol Oncol. 2020 Jul 27;13:103. doi: 10.1186/s13045-020-00924-z (PMC7384229; doi:10.1186/s13045-020-00924-z)

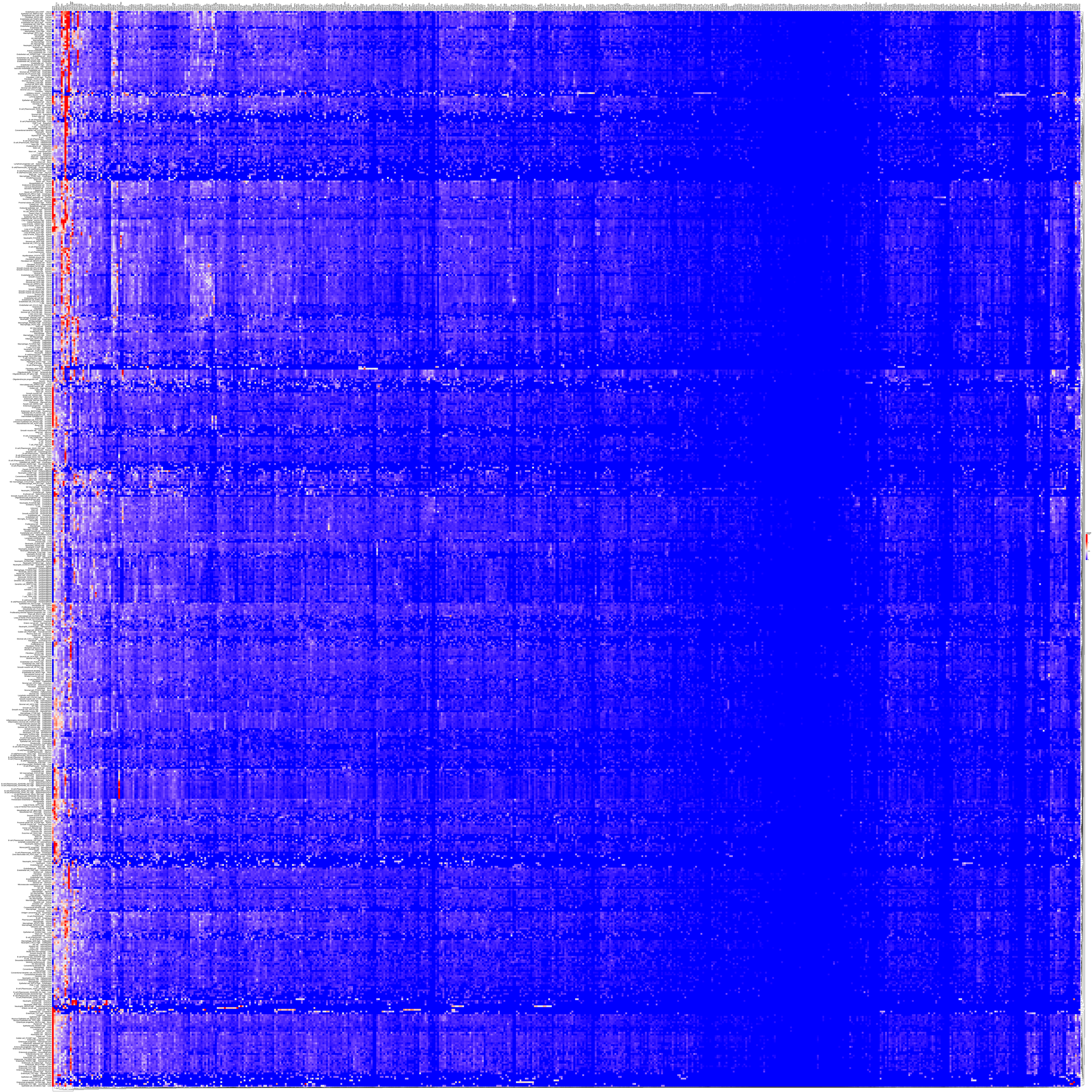

Supplement: Supplementary file 1 — Additional file 1. Profiling E3 ligase expression at single cell level in different tissues. The heatmap shows the single-cell expression levels of 574 E3 ligases across all cell types and tissues in human adults from the Human Cell Landscape (HCL) data. In the heatmap, each row represents a cell type, and each column represents an E3 ligase. The color legend was shown on the right, which was determined by averaging the expression values of all cells within a tissue-specific cell type. These E3 ligases and the different cell types were clustered by the hierarchical clustering algorithm, and the resulting dendrograms were shown on bottom and right, respectively. [file 13045_2020_924_MOESM1_ESM.pdf]
